# Supplementary material for: Becoming more of an insider: A grounded theory study on patients’ experience of a person-centred e-health intervention
Source: PLoS One. 2020 Nov 23;15(11):e0241801. doi: 10.1371/journal.pone.0241801 (PMC7682879; doi:10.1371/journal.pone.0241801)
Supplement: S1 Fig — (PDF) [file pone.0241801.s001.pdf]

Interview guide

Starting question

"Can you please tell me about your thoughts when you found out that you were offered person-centered telephone calls and access to a digital platform?"

Question areas

My Health digital platform (content and design)

- Links
- Health plan
- Daily ratings
- Inviting family or friends
- Introduction/Education about the digital platform

- Phone calls/conversations
- Meeting health care professionals remotely
- Type of technical aid (computer, tablet, smart phone)
- Barriers and facilitators
- Time perspective
- Similarities and differences with previous care experiences
- Future perspectives on digital care services
- Need of support or not*
- Support vs help*
- Roles in keeping track, own or others (self-together)*
- Individual process*
- Individual prerequisites*
- Target group and utilization of digital platform*
- Somebody cares*

*Question areas added in line with theoretical sampling

Ending questions

If you were to tell someone who has not been participating about the person-centered phone calls and access to the digital platform, what would you say?

Is there anything you would like to tell me that we have not talked about?

Is there anything else you think I should know to understand?

Examples of intermediate questions that could be used to capture the process

What did you know before the "intervention" about what…? (…appeared in dialogue during the conversations, was read on the platform, etc.).

Has your view of X changed when using the E-health support? Can you tell me in what way?

Can you please describe what made it change?
