# Supplementary material for: Becoming more of an insider: A grounded theory study on patients’ experience of a person-centred e-health intervention
Source: PLoS One. 2020 Nov 23;15(11):e0241801. doi: 10.1371/journal.pone.0241801 (PMC7682879; doi:10.1371/journal.pone.0241801)
Supplement: S2 Fig — (PDF) [file pone.0241801.s002.pdf]

Frågeguide svenska

Inledande fråga

Vill du berätta vad du tänkte när du fick reda på att du var en av dem som skulle få tillgång till personcentrerade samtal och den digitala plattformen?

Frågeområden

- Min Hälsa plattform (Innehåll + Utformning)
  - Länkar
  - Hälsoplan
  - Dagsform
  - Bjuda in partners
  - Introduktion/utbildning portalen
- Samtal
- Möta personal på distans
- Medium/Använt medium (Dator, surfplatta, smartphone)
- Hinder & Möjligheter (förutsättningar)
- Tidsperspektiv
- Likheter & skillnader med tidigare vårderfarenheter
- Framtid (Hur går vi vidare?)
- Målgrupp och användningsområde*
- Egna förutsättningar*
- Egen process*
- Någon bryr sig*
- Behov av ”insats”/stöd – inget behov*
- Stöd kontra Hjälp*
- Egen kontra olika partners roll (i att hålla koll) (själv-tillsammans)*

Avrundande frågor

Om du skulle berätta om E-hälsastödet (Min hälsa portal samt personcentrerade samtal) för någon som inte har varit med vad skulle du säga då?

Är det någonting du skulle vilja berätta som vi inte har pratat om?

Är det någonting annat du tycker att jag bör veta för att

Exempel på frågor som stöd för att fånga processer

Vad visste du innan ”interventionen” om det som…? (framkom i dialog under samtalen, fanns att läsa på plattformen osv.).

Har din syn på X ändrats vid användning av E-hälsostödet?

Kan du berätta för mig på vilket sätt?

Kan du beskriva för mig vad det är som har gjort att det har förändrats?

*tillagda frågeområden i linje med teoretisk sampling
