# Supplementary material for: Becoming more of an insider: A grounded theory study on patients’ experience of a person-centred e-health intervention
Source: PLoS One. 2020 Nov 23;15(11):e0241801. doi: 10.1371/journal.pone.0241801 (PMC7682879; doi:10.1371/journal.pone.0241801)
Supplement: Attachment — Submitted filename: Plos One 2020.docx [file pone.0241801.s003.docx]

Article: PEC-20-841, "Specific Healthcare Responsibilities and Perceived Transition Readiness Among Adolescent Solid Organ Transplant Recipients"

This is a well written and important article which aims to explore the experiences of a person-centred e-health intervention in patients diagnosed with chronic obstructive pulmonary disease (COPD) or chronic heart failure (CHF).

Before considering publication, I have some questions which need to be clarified.

**The intervention**

The number of phone calls was “determined based on an agreement between the patient and the health professionals”. Was there, however, a range of conversations offered?

The table 1 is very informative concerning the platform and its use and accesses.

**Sampling**

It is not quite clear how the theoretical sampling which is considered a major part of grounded theory has taken place. In the abstract you write that twelve patients were “purposefully sampled in accordance with the initial sampling criteria”.

I read “the initial sampling criteria” as the sampling criteria used in the PROTECT study which is presented as an RCT study. Thus, patients were sampled among patients in the intervention group. Yet, sampling could still be made theoretical where constant comparison of the initial findings from the first sampled patients could inform the further sampling. I wonder whether this has taken place. On the other hand, at page 8 you write that “data collection and analysis were done concurrently”. Please clarify if theoretical sampling only has been done by as mentioned at page 7 “developing the interview guide in line with theoretical sampling.

Moreover, when were the patients asked to participate? How many were asked and did all agree?

**Results**

The grounded theory “Being welcomed through the side door when lacking the front door keys to protect health” is clearly developed and based on data in the form of citations. Sometimes the context needs to be better explained. For instance, the first quote at page 10, *gained some hope that this* [what does “this” refer to?] Please elaborate on the context or explain in the [ ].

The second quote at page 10. What does “ball blank” mean in the sentence *“why people have a ball blank to bounce ideas off”*?

Overall the theory provides a deeper and meaningful understanding of the advantages experienced by patients having a remote intervention which is person-centered and at the same time e-based. Especially the flexibility in patients’ ability to choose when to be active and when NOT to be active in the intervention was very convincing.

The figure gathers the theory well and is well placed at page 9.

**Discussion**

A well written discussion is provided which both discuss importance of the findings and acknowledges study limitations and how they were addressed. The implications of how the study can inform evaluation of the intervention in the overall study is also explained. Literature in the field is included satisfactorily.

Minor comment

Page 4 Standards for Reporting Qualitative Research should be written before the abbreviation “SRQR”.
